# Guiding antiferromagnetic transitions in Ca$_{2}$RuO$_{4}$

**DOI:** 10.1038/s41598-022-14932-1

**Published:** 2022-06-29

**Authors:** D. G. Porter, F. Forte, V. Granata, M. Cannavacciuolo, R. Fittipaldi, M. Cuoco, A. Bombardi, A. Vecchione

**Affiliations:** 1 Diamond Light Source Ltd., Harwell Science and Innovation Campus, Didcot, Oxfordshire OX11 0DE UK; 2 CNR-SPIN, c/o Universitá di Salerno-Via Giovanni Paolo II, 132-84084 Fisciano, SA Italy; 3 Dipartimento di Fisica ‘E.R. Caianiello’, Universitá di Salerno, 84084 Fisciano, Salerno Italy; 4 Department of Physics, University of Oxford, Parks Road, Oxford, OX1 3PU UK

**Keywords:** Magnetic properties and materials, Electronic properties and materials, Phase transitions and critical phenomena

## Abstract

Understanding and controlling the transition between antiferromagnetic states having different symmetry content with respect to time-inversion and space-group operations are fundamental challenges for the design of magnetic phases with topologically nontrivial character. Here, we consider a paradigmatic antiferromagnetic oxide insulator, Ca$_{2}$RuO$_{4}$, with symmetrically distinct magnetic ground states and unveil a novel path to guide the transition between them. The magnetic changeover results from structural and orbital reconstruction at the transition metal site that in turn arise as a consequence of substitutional doping. By means of resonant X-ray diffraction we track the evolution of the structural, magnetic, and orbital degrees of freedom for Mn doped Ca$_{2}$RuO$_{4}$ to demonstrate the mechanisms which drive the antiferromagnetic transition. While our analysis focuses on a specific case of substitution, we show that any perturbation that can impact in a similar way on the crystal structure, by reconstructing the induced spin–orbital exchange, is able to drive the antiferromagnetic reorganization.

## Introduction

In materials that exhibit antiferromagnetism, the magnetic moments of atoms align in a regular pattern with neighboring spins, on different sublattices, orienting along different directions to achieve zero net moment. Frustration can arise from the impossibility of having all interactions favourable and often results in the formation of competing degenerate energy states. Apart from geometrical aspects of the crystal lattice, degeneracy in the ground state manifold or in the spectrum of excitations can be dictated by the way time-reversal symmetry combines with crystal point group transformations. A typical example is represented by antiferromagnets with a magnetic space group where point group and time-reversal-symmetry operations are combined with fractional lattice translations, but neither space-group nor time-reversal operations are lone symmetries. This circumstance can occur for instance when lattice sites that locally exhibit time-reversal symmetry breaking have time-reversed partners elsewhere in the unit cell. Recently, it has been pointed out that topological phases, insulating or semimetallic, are possible in antiferromagnets even though time-reversal symmetry is broken^1–5^. These studies have led to the prediction of Dirac antiferromagnets and topological insulating phases with the promise to set out novel paradigms for spintronics devices. Since the topological character of a quantum phase is strongly tied to the symmetry and dimensionality, understanding and controlling the transition between symmetrically distinct antiferromagnetic (AFM) ground states is fundamental milestones for unveiling and exploiting emergent magnetic topological phases.

In this work we tackle this challenge by considering an emblematic AFM oxide insulator, Ca$_{2}$RuO$_{4}$ (CRO), that exhibits two possible magnetic structures, close in energy, and related only by a local breaking of time-reversal symmetry on a nonsymmorphic symmetry element—a screw-axis or glide-plane operation^6–8^. We show that the transition between these phases can be generally obtained by orbital reconstruction through substitutional doping. Orbital degrees of freedom are known to set out the character of the magnetic exchange in complex oxides. Here, the modified magnetic exchange and the structural distortions driven by the substitution cooperate to yield a variation of the orbital occupancy that in turn controls the changeover of the AFM patterns.

**Figure 1:**
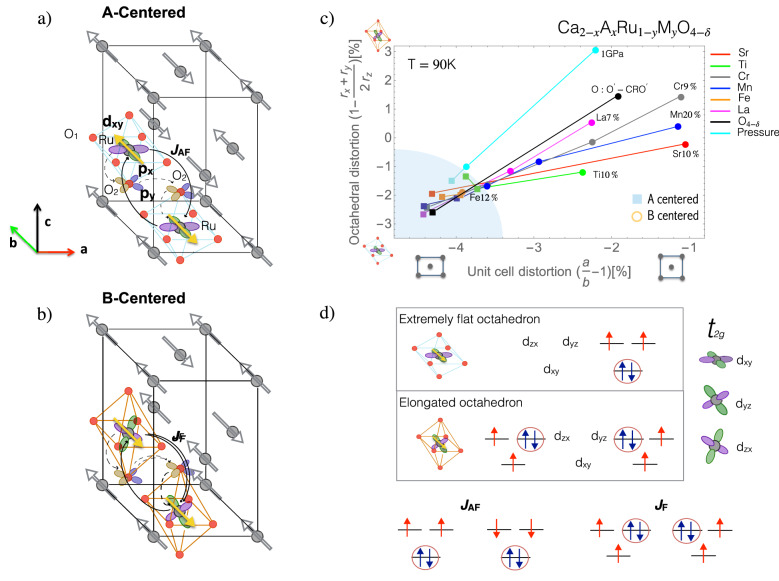
Schematics of the magnetic structures reported in pure and Mn-substituted CRO. The A-centered (**a**) and B-centered phase (**b**) differ in the stacking sequence of adjacent layers, with moments of the intermediate ruthenium layers pointing in opposite direction. Magnetic superexchange paths are highlighted between neighboring ruthenium ions along the *c*-axis, passing two oxygen ions. For simplicity, the small canting of magnetic moments along the *c*-axis and tilting and rotation of the RuO$_{6}$ octahedra are not displayed. (**c**) Variation in octahedral distortion to unit cell distortion across different structure alteration regimes, for structure data of CRO from the literature related to several dopings, pressure and changes in oxygen stoichiometry. The octahedral distortion is calculated from RuO$_{1}(r_{x}, r_{y})$ and RuO$_{2}(r_{z})$ bond lengths. All structures showed are at 90 K. Where structure data is not available at 90 K, it is estimated based on available temperatures, using the variation in temperature observed in the pure compound^8,12,14–20^. The magnetic state is given as determined by direct methods, or inferred through susceptibility measurements^8,12,15,16^. (**d**) Left side: dominant AFM exchange between Ru spin moments, in doubly occupied $d_{xy}$ orbital configuration favored by the compressive tetragonal distortion of the pure CRO. Right side: dominant FM exchange between Ru spins in the alternated doubly occupied $d_{zx}$/$d_{yz}$ orbital configuration induced by substitution.

CRO is the n = 1 end member of the Ruddlesden–Popper family of ruthenium oxides and can be distinguished from its superconducting sibling Sr$_{2}$RuO$_{4}$ by the lower symmetry $Pbca$ structure due to the significant distortions of the RuO$_{6}$ octahedra caused by the size effect arising from reduced Ca atomic radius. At lower temperature an onset of orbital ordering has been reported at T$_{OO}$ = 260 K, though the exact nature of this phenomena remains unclear^9–11^. The system orders antiferromagnetically at T$_{N}$ = 110 K, with moments predominantly directed along the b-axis. The ground state of CRO is prone to magnetic reconstruction upon perturbations, as distortions of the structure generally result in a changeover from the A-centered phase to the B-centered one^8,12–17^, illustrated in Fig. 1a and b, respectively. These magnetic structures differ by a change in relative orientation of the z = 0.5 layer. The B-centered phase has a magnetic space group of $Pb'c'a$ (compared with *Pbca* for the A-centered phase) with time inversion appearing on screw axes along the b and c axes, allowing in this case a collective ferromagnetic (FM) moment along the a-axis and a higher ordering temperature.

In Fig. 1c we exhibit structural data from the wide literature on this material and including our own data on pure CRO and this work on Ca$_{2}$Ru$_{1-x}$Mn$_{x}$O$_{4}$, covering a variety of different doping scenarios and consequences of pressure or oxygen changes in the stoichiometry. We unveil a general trend concerning the interplay of structural and magnetic properties: the A-centered phase is only found under the most extreme negative distortions of both the cell and the octahedra, i.e. when the RuO$_{6}$ octahedra are more flat and the cell is most elongated along the b-direction—a situation that only occurs close to the pristine compound. The magnetic reconstruction can be understood by considering that the modification of the orbital occupation at the Ru site alters the character of the Ru–Ru magnetic exchange from antiferromagnetic to ferromagnetic, as schematically described in Fig. 1d, with the magnetic exchange path illustrated in Fig. 1a. This has a crucial role to pinpoint the antiferromagnetic configuration when considering the inter-layer coupling of Ru spins (Fig. 1a,b). We point out that such correlation-driven reconstruction of the orbital ordering is compatible with local structural distortions where the octahedra are turned from flat to elongated. In this respect, the similarity in the octahedral distortions of CRO under different scenarios, as for example changing the ionic radius of the cation (Ca > La or Sr), the oxidation state or applying pressure, allows to harmonize and account for all the observations of the phase diagram in Fig. 1b. Along this line, here we focus on doping the ruthenium site with manganese–a magnetic transition metal with a similar radius but one less electron in valence. This is a paradigmatic example for carefully tracking the change in phases and validate the mechanisms behind the antiferromagnetic reconstruction.

## Experimental results

The presence of nonsymmorphic symmetry elements in a space group, i.e. glide planes or screw axes, is always associated with destructive interference that leads to serial absences of reflections related to the nonsymmorphic elements in a standard X-ray experiment (See details of the nonsymmorphic transformations in Sec. A of the Supplementary Information online).

In the resonant condition, when the photon energy is tuned to an electronic transition from a core state to the outer states involved with cooperative ordering, these reflections are present and provide a unique probe of charge, orbital or magnetic ordering that violate the serial extinction rules^21^.

CRO presents three perpendicular glide planes and in this experiment we used two classes of reflection associated to two of them (0, 2n + 1, l) and (2n + 1, 0, l) to probe the A-centered and B-centered magnetic structure^11^. Assuming that the ordered magnetic moments are almost confined along the b axis as consequence of the large single ion anisotropy present in these materials, depending on the relative alignment between the z = 0 and the the z = 1/2 layer, reflection of the first or of the second type are activated when the photon energy is tuned close to the Ru L$_{2}$ or L$_{3}$ absorption edges.

**Figure 2:**
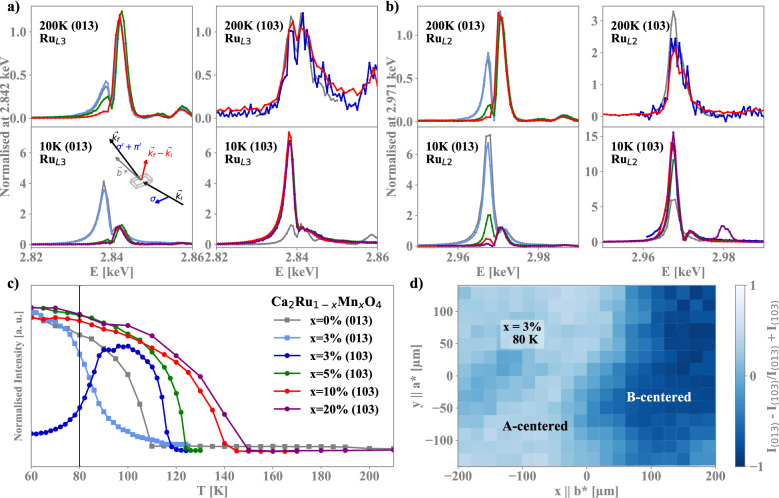
Resonant X-ray spectra at the Ru-L3 (**a**) and Ru-L2 (**b**) edges on two charge-forbidden reflections. In each case the polarisation azimuth is chosen such that the b* axis is parallel to the beam with $\sigma$ incident polarisation, as shown in the inset of (**a**). Below the transition (10 K), the two reflections are dominated by resonant magnetic scattering from the A-phase (013), or the B-phase (103). Above the transition (200 K), the remaining resonant signal is sensitive to the orbital occupation and the variation with Mn concentration indicates a shift in orbital population. Where spectra is missing for certain concentrations, the reflection was not observable. (**c**) The temperature dependence of the resonant reflections highlights the variation of the magnetic transition with concentration and the switch between the A and B magnetic phase in the 3% sample. (**d**) The variation in reflection intensity of the two reflections at 80 K across the surface of the 3% sample, indicating the presence of magnetic domains of the two magnetic phases.

In Fig. 2a we summarize the evolution of the magnetic ordering as a function of the Mn content, as deduced by the L$_{3}$ resonant behavior measured at 10 K (Fig. 2a bottom). Both figures are dominated by the signal next to 2.838 keV, associated with the $t_{2g}$ multiplet responsible for the magnetic ordering. The $e_{g}$ states (at 2.842 keV) are largely unaffected by the magnetic ordering, were used for renormalization to allow for the comparison between different samples (Resonant X-ray Spectra of each reflection without renormalization by the $e_{g}$ states are included in Sec. B of the Supplementary Information online). In the case of pure CRO at 10 K a large signal is present on top of the (013) reflection, whereas almost no variation compared to the paramagnetic signal is present on top of the (103) reflection, as expected in the case of an A-centered magnetic ordering.

For all the Mn concentration above 3% we found a reversed situation, with a large enhancement present on top of the (103) reflection and almost no enhancement on top of the (013), clearly indicating that the B-centered magnetic ordering was realised in these compounds. The collective ferromagnetic moment associated with canting in the a-axis of the B-centered ordering explains an increase in magnetisation below the transition temperature observed in magnetisation measurements of the doped samples. Magnetisation measurements presented in Sec. C of the Supplementary Information online. The canting associated with this FM moment is not visible in these measurements due to the large background from charge scattering at reflections sensitive to this moment. A small moment along the c-axis has been reported in pure CRO and some evidence of a similar canting above 3% can is found with a small enhancement of the (013) reflection. In the 3% system (light blue curve) the two magnetic orderings coexists, and a large enhancement is present on forbidden reflections.

The thermal evolution of the (013) and (103) measured on top of the $t_{2g}$ multiplet (E = 2.838 keV) presented in Fig. 2c confirms the different magnetic ordering, whereas the change in ordering temperature from the A to the B phase provides an estimate of the variation in the global strength of the effective exchange couplings. In the 3% system, where the two magnetic orderings are nearly degenerate and closely competing, above 90 K the system tends to prefer the B-centered magnetic structure but the A-centered structure is already present and slowly grows with reducing the temperature. Below 90 K, the A-centered structure dominates the magnetic landscape. A sample map at 80 K with a $20\times 20$ μm^2^  resolution confirmed the presence of both phases in the whole crystal but with a change of relative weight across different areas (Fig. 2d), whose origin is not associated with any variation in the Mn content, as Scanning Electron Microscope measurements revealed that the Mn content is homogeneous across the same sample with an accuracy of 10% of the nominal concentration. Such a situation could appear, however, due to local formation of B-centered structure at high temperature, which then persists below the transition to the A-centred phase due to domain formation via the FM field exerted by canting in the B-centered structure.

It is worth noticing that the reflections (0, 2n + 1, l) and (2n + 1, 0, l) are present also in the paramagnetic phase^11^. There (Fig. 2b top), in absence of the dominating signal related to magnetic ordering, their intensity can be associated to the magnitude of the asymmetry present in the *d*-states charge distribution. Given the large angle formed by the the (013) and the (103) reflections with the *c* axis and the tilting of the RuO$_{6}$ octahedra, these reflections probe the whole $t_{2g}$ subspace with different weights quantified in ref.^11^.

The comparison of the spectra with increasing the Mn content reveals a significant jump in the intensity associated with the $t_{2g}$ multiplet related to the reflection (013) (Fig. 2b top left), with the two samples that exhibit a transition toward the A-type magnetic phase (0 and 3%) at $T_{N}$ showing a much larger feature. This pervasive feature, also visible at the L$_{3}$ edge, can be associated with the local spin orbit coupling modifying the electronic distribution of the $t_{2g}$ states and altering the specific density of states projection seen in resonant condition on the 0 and 3% compared with the 5 and 10% samples. This behavior provides experimental support to the electronic changes suggested in the $t_{2g}$ levels and can be related to the structural changes occurring in the doped samples and presented in Fig. 1c.

These structural changes are well established in pure CRO^8^ and have also been reported for a range of different doping regimes highlighted in Fig. 1c. In the Mn doping case, our laboratory X-ray measurements of the evolution of the atomic structure with temperature are reported in Sec. D of the Supplementary Information online. As with other Ruddlesden–Popper based systems, CRO shows a still not fully understood uniaxial negative thermal expansion, in this case along the b-axis, accompanied by rotation, tilting and flattening of the octahedra under cooling that does not change the space group symmetry of the system. When Ru is replaced by Mn, the substitution moderates the structural distortion at the MIT and it reduces the flattening of the octahedra, primarily induced by a reduction in the planar RuO$_{1}$ bond length and reduced octahedral rotation. This leads to decreased paths between neighboring Ru ions in all directions except between planes along the a-axis, which sees a slight increase on the Ru–O$_{2}$–O$_{2}$–Ru(½,0,½) path through the apical O$_{2}$ oxygen.

## Theoretical analysis

The experimental analysis revealed that the A-centered magnetic phase is only achieved under the most extreme flattening of the RuO$_{6}$ octahedra, and orthorhombic deformation of the unit cell, with values of the ratio $\frac{a}{b}<$1. Moreover, both the structural and magnetic characterization, together with the Resonant X-ray diffraction study, show that a minute percentage of Mn impurity doping yields an orbital reconstruction of the $t_{2g}$ levels, which occurs together with the transition among the two close in energy A- and B-centered magnetic structures.

Several studies have analyzed the exact nature of the magnetic ground state of pure CRO. It is now well established that a robust in-plane AF order takes place in the RuO layers, accompanied by an *xy*-polarized orbital order due to the extreme flattening of the RuO$_{6}$ octahedra, as also confirmed by experiments^9–11^. However, there is still a lack of studies that examine the nature of the interlayer magnetic coupling, as well as the possibility to tune from the A- to the B-centered structures belonging to distinct magnetic subgroups. A fundamental aspect emerging from our work is that the orthorhombic deformation helps to release the frustration of the out-of plane magnetic exchange, by making the path lengths between neighbouring Ru ions along the *a* and *b* axis quite different. The shorter magnetic exchange path in the (*a*, *c*) plane is crucial in favoring the A phase over the B phase.

In the following, we aim to unveil the microscopic mechanism underlying the stabilization of the B-centered magnetic structure in the CRO via a very low concentration of Mn impurities. In order to connect the evidence of our experimental data with a microscopic physical picture, we employed a microscopic model which includes all interaction terms at the ruthenium and oxygen sites and the kinetic term for the ruthenium–oxygen connectivity within the (*a*, *c*) plane (For details, see Sec. E of the Supplementary Information online^22,23^.

**Figure 3:**
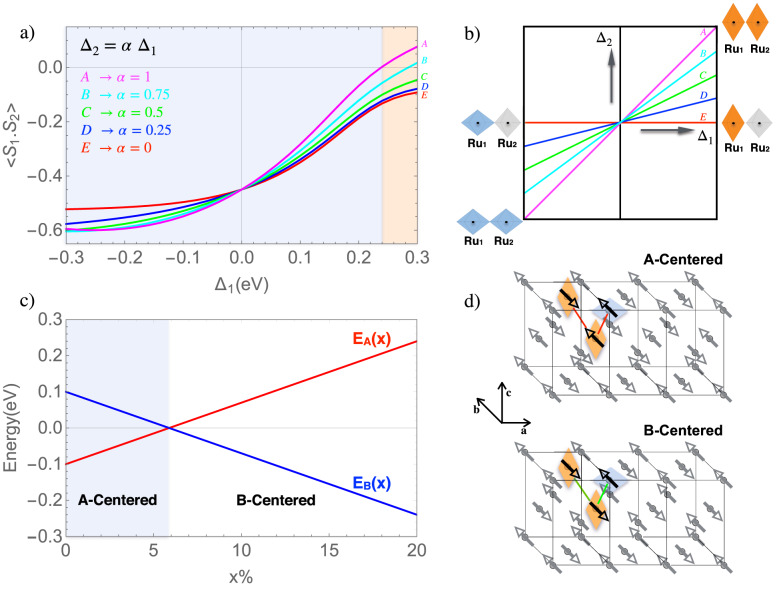
(**a**) Theoretical model calculation of the spatial spin correlations developing among two Ru ions linked along the $(\frac{1}{2} ,0,\frac{1}{2} )$ direction. Different curves refer to different conditions for orbital reconstruction around the Mn-impurity sites, corresponding to several values of the ratio $\alpha$ between the tetragonal crystal field terms $\Delta _{1}$ and $\Delta _{2}$, as depicted in panel (**b**). The orange shaded area corresponds to the region where one gets the changeover form AF to FM bond exchange. Panel (**d**) shows a schematic view of the magnetic patterns A and B, where the orbital reconstruction around the impurity sites induced by $d^{3}$ impurity in a $d^{4}$ host is highlighted. In particular, Blue (orange) colors indicate compressed (elongated) octahedra respectively. We notice that in the B-centered structure, an FM exchange gain along the (**a**,**c**) as well as of the AFM exchange gain along the (bcc) (green lined) are allowed. Panel (**c**) shows the comparison of the energies of the A and B phase, as a function of the Mn impurity concentration x.

As for the effect due to Mn impurities, it is taken into account as follows. Doping with Mn$^{4+}$ impurities leads to substitution of $d^{4}$ with $d^{3}$ configurations, having local S = 3/2 and without an orbital degree of freedom (doublon). It has been recently demonstrated that the $d^{4}$–$d^{3}$ spin–orbital exchange driven by Coulomb correlations yields an orbital reconstruction around the impurity sites^24,25^. In particular it acts as an effective crystal field potential $\Delta$, which tends to promote the doublon to the ($d_{zx,}$, $d_{yz}$) sector (see Fig. 3b). In CRO, this impurity exchange tends to compete with the lattice potential, and can even end up reversing the sign of the crystal field interaction. Within this frame, we include in our model an additional effective term, which simulates the inversion of the crystal field (CF) at the sites located around the impurity. We investigate the microscopic model by means of exact diagonalization (ED) on a cluster consisting of a Ru–O$_{2}$–O$_{2}$–Ru arrangement of transition metal and oxygen ions, in a fashion which reproduces the connectivity within the (*a*, *c*) plane. We determine the most favored magnetic exchange via the calculation of the static structure factor (SSF) among the Ru spins, and the way it gets modified by the effect induced by Mn doping on the orbital population at the Ru sites.

In Fig. 3 we report the evolution of the SSF as a function of the effective CF terms at the Ru sites $\Delta _{1}$ and $\Delta _{2}$. The parametrization $\Delta _{2}$ = $\alpha \Delta _{1}$ allows to take into account the possibility of inhomogeneous reconstruction of the orbital population on the two sites, as schematically depicted in panel (b). Extreme negative values of $\Delta _{1}$ corresponds to the undoped CRO compound where the *xy* orbital is about fully occupied. The magnetic exchange in this condition is schematized in Fig. 1d, where we show the effective AFM interaction between the Ru magnetic moments, in an orbital configuration where the *zx* and *yz* are half filled and form a spin one.

Increasing $\Delta _{1}$ gives rise to a crossover from dominant in-plane AFM correlation to FM, notably this tendency being enhanced in the case of homogeneous deformation $\alpha =1$. Such a scheme is represented in Fig. 1d, where we show the effective FM exchange interaction between the Ru magnetic moments localized at the Ru sites, in an orbital configuration where the doublon occupies the *zx* and *yz* in an alternated way.

Next we used the estimates of the exchange parameters $J_{AF}$ and $J_{F}$ extracted from the cluster calculation, to compute the energy of the A & B phases and determine the most stable one. In particular, we consider the way the total energy E$_{\alpha }$ ($\alpha$ = A, B) gets modified due to the introduction of a very small number of orbital defects, as schematized in Fig. 3d). Here, we highlight the bonds where the favored exchange is altered, because of the inverted CF due to very diluted Mn impurities. Both the magnetic exchange along the short and long exchange path are frustrated (red line) in the A-centered structure, while they give rise to an extra energy gain in the B-centered phase. In Fig. 3c we plot the evolution of E$_{\alpha }$ as a function of the increased Mn concentration *x*. Such behavior shows that even a tiny concentration is capable to favor the magnetic B-centered over the A-centered. In conclusion, we can state that small perturbation of the orbital density due to the impurities, modifies the nature of the magnetic exchange in the (*a*, *c*) plane from AFM to FM, and consequently, even a very low concentration, around 5 $\%$ may allow the transition from one to the other.

## Discussion

The fate of magnetic ordering in oxides is generally dictated by the spin–orbital exchange among transition metal elements. The character of the magnetic exchange depends on the occupation of the *d*-states at the transition metal site and on the corresponding orbital configuration. When electrons localize, the latter is typically influenced by the presence of non-negligible structural distortions and spin–orbit coupling. In absence of spin–orbital frustration, the Goodenough-Kanamori rules indicate that antiferromagnetism with ferro-type orbital configuration or ferromagnetism with alternated orbital pattern are expected to occur, respectively^26,27^.

Here, the CRO provides a completely novel paradigm because it allows us to demonstrate that it is possible to tune among competing antiferromagnetic phases by reconstructing the interlayer spin–orbital exchange via substitutional doping of the transition metal. CRO is an insulator for which doping through the substitution of the transition metal element acts to alter the spin–orbital correlations, without destroying the insulating state. Remarkably, the mechanism controlling the transition among different types of antiferromagnetic phases exploits the orbital degrees of freedom but it does not depend on further breaking symmetry or crystal point group symmetry reductions. The observed behavior is thus expected to be general in nature also due to the firm relation experimentally established between the precursory electronic orbital arrangement observed in the paramagnetic phase and the selected magnetically ordered ground state.

The reconstruction of the magnetic exchange along the inter-layer direction is crucial to unbalance the energy competition of the two competing antiferromagnetic phases. This mechanism is thus expected to be applicable for all layered antiferromagnetic systems exhibiting competing phases along nonsymmorphic symmetry directions. There are two relevant and unique hallmarks of the unveiled mechanism. Firstly, it has an impact on a short range length scale with consequences on the long range magnetic ordering. As we have demonstrated in the theoretical analysis, it is enough to have a very small quantity of defects through the material where the reversal of the sign of the inter-layer exchange occurs to guide the antiferromagnetic reconstruction. The second aspect refers to the role of the orbital degrees of freedom. We demonstrate that the sign change of the magnetic exchange is pinned to the modification of the orbital occupation at the transition metal element and is related to the intrinsic orbital anisotropy of the material upon examination. For instance, a variation of the crystal field potential close to the defects can directly promote the filling of out-of-plane orbitals. This orbital dependence indicates that the transition can be obtained either via the control of the lattice or of the electronic degrees of freedom by suitably tuning the orbital occupancy. In this context, the substitutional doping of Mn is a paradigmatic example where both lattice and electronic degrees of freedom cooperate to reconfigure the orbital occupation close to the defects.

## Conclusions

Through the substitutional doping of Mn in Ca$_{2}$RuO$_{4}$ we have found through use of X-ray scattering techniques the ability to control and measure the subtle change in magnetic ordering induced without changing the symmetry of the crystal structure. RXS measurements allow us to unambiguously determine the magnetic ordering and separate the change in orbital population from structural changes observed through traditional X-ray diffraction measurements. We present a mechanism to explain this transition that requires both the crystal field and spin–orbit coupling effects. The resulting mechanism is therefore general and helps to understand the universality of the change from the A-centered to B-centered magnetic phase in the material under a wide range of different doping strategies and also provides a general explanation for removing degeneracies along such nonsymmorphic symmetry elements.

Since the transition can be induced by means of atomic substitution, our results set out new paths for the design of magnetic phases by dopants engineering or, in perspective, by exploiting surface deposition in ultrathin films to induce magnetic surface reconstructions. Moreover, the possibility of achieving a mechanism to control magnetic properties through nonsymmorphic symmetry elements paves the way to novel materials design with topological antiferromagnetic phases, accompanied by spin–orbital excitations that can be confined at the edge between A and B magnetic domains and in general robust to perturbations due to symmetry based topological protection.

## Methods

Single crystals of Ca$_{2}$Ru$_{1-x}$Mn$_{x}$O$_{4}$ with *x* = 0, 0.03, 0.05, 0.10, 0.20 were grown using a floating zone furnace and characterised prior to synchrotron beamtime. The challenge of growing large crystals of Ca$_{2}$Ru$_{1-x}$Mn$_{x}$O$_{4}$ is related to the abrupt structural transition occurring close to 350K, however we found that cooling slowly through the transition resulted in large plate shaped samples with the (00L) direction perpendicular to the large face and dimensions $\approx 1000\times 1000\times 100$ $\upmu {\hbox {m}}^{3}$. Compositions with increased Mn content tended to be larger but with less regular shape and a larger number of crystalline grains. Structural characterisation was performed using a molybdenum source X-ray diffractometer (Oxford Diffraction) with a cryojet (Oxford Instruments) for temperature control between 90 and 500 K. Magnetisation measurements were performed using the DC measurement option on an Quantum Design MPMS3. In each case the sample was aligned prior to measurement with X-ray diffraction and mounted to a quartz rod with GE varnish such that the c-axis was perpendicular to the field and either the *a*-or-*b* axis were parallel to the field. Samples were measured in on cooling in a field of 1000 Oe (field-cool).

Resonant X-ray scattering (RXS) measurements were performed at beamline I16, Diamond Light Source Ltd.^28^ on single grain samples of each composition. Samples were attached to the beamline cryostat (ARS closed cycle cryocooler), with a copper mount and silver paint to ensure good thermal contact. The beamline optics were tuned to the ruthenium $L_{3}$ absorption edge (2.828 keV) by performing a four bounces on the Si(111) monochromator, ensuring high flux and good focus in this tender X-ray range. An additional set of silicon mirrors were used to eliminate harmonic contamination. At this energy, the focused spot size was $\approx 180\times 50$ $\upmu {\hbox {m}}^{2}$. The air path of the incident and scattered X-ray beam was reduced to $\approx$4 cm by use of in-vacuum detectors, with only SiN and Kapton X-ray windows, plus a single Be cryostat dome in the beam. Measurements were made with an in-vacuum Pilatus-3 (Dectris) area detector and the scattered beam polarisation was analysed with a highly oriented graphite plate aligned to measure the (002) reflection with an avalanche photo-diode at $\approx$ 90$^\circ$ to the scattered beam. The cross-channel leakage of the analyser crystal at this energy was < 5%. In all cases the azimuthal zero angle was defined where the (010) crystal axis intersects the scattering plane in the forward scattering direction and a positive rotation is anti-clockwise about the wavevector transfer (${\vec {k}}_{f}$–${\vec {k}}_{i}$).

## Supplementary Information

Supplementary Information.

## Data Availability

The datasets generated and/or analysed during the current study are available in the Zenodo repository, 10.5281/zenodo.6497537.
