# Supplementary material for: Guiding antiferromagnetic transitions in Ca2RuO4
Source: Sci Rep. 2022 Jun 29;12:10957. doi: 10.1038/s41598-022-14932-1 (PMC9242999; doi:10.1038/s41598-022-14932-1)
Supplement: Supplementary file 1 — Supplementary Information. [file 41598_2022_14932_MOESM1_ESM.pdf]

Guiding antiferromagnetic transitions in Ca_2RuO_4

D. G. Porter[1,*,+], F. Forte[2,3,+], V. Granata[3], M. Cannavacciuolo[3], R. Fittipaldi[2,3], M. Cuoco[2,3,], A. Bombardi[1,4], and A. Vecchione[2,3]**

[1]Diamond Light Source Ltd., Harwell Science and Innovation Campus, Didcot, Oxfordshire, OX11 0DE, UK
[2]CNR-SPIN, c/o Universitá di Salerno- Via Giovanni Paolo II, 132 - 84084 - Fisciano (SA), Italy
[3]Dipartimento di Fisica 'E.R. Caianiello', Universitá di Salerno, Fisciano, Salerno I-84084, Italy
[4]Department of Physics, University of Oxford, Parks Road, Oxford OX1 3PU, United Kingdom
[*]dan.porter@diamond.ac.uk
[**]mario.cuoco@spin.cnr.it
[+]these authors contributed equally to this work

ABSTRACT

Understanding and controlling the transition between antiferromagnetic states having different symmetry content with respect to time-inversion and space-group operations are fundamental challenges for the design of magnetic phases with topologically nontrivial character. Here, we consider a paradigmatic antiferromagnetic oxide insulator, Ca_2RuO_4, with symmetrically distinct magnetic ground states and unveil a novel path to guide the transition between them. The magnetic changeover results from structural and orbital reconstruction at the transition metal site that in turn arise as a consequence of substitutional doping. By means of resonant x-ray diffraction we track the evolution of the structural, magnetic, and orbital degrees of freedom for Mn doped Ca_2RuO_4 to demonstrate the mechanisms which drive the antiferromagnetic transition. While our analysis focuses on a specific case of substitution, we show that any perturbation that can impact in a similar way on the crystal structure, by reconstructing the induced spin-orbital exchange, is able to drive the antiferromagnetic reorganization.

Supplementary Information

A Symmetry Operations in Pbca magnetic space groups

In Fig. S1 we illustrate the glide plane, screw axis and time operations between the central Ru ion at (0,0,0) and the neighbours in the next layer - Ru(1/2,0,1/2) and Ru(0,1/2,1/2).

B Resonant X-Ray Diffraction

We have previously reported on the resonant behaviour of the un-doped case of Ca_2RuO_4 at the ruthenium L-edges, where we found that resonant reflections are found at the serial extinctions and that these can be collected into three classes, each sensitive to moments along different axes depending on the magnetic ordering[1], shown below in Table S1.

	$k+l$ =even<br>$h+l$ =odd<br>e.g. (013), (100)	$k+l$ =odd<br>$h+l$ =even<br>e.g. (103), (010)	$k+l$ =odd<br>$h+l$ =odd<br>e.g. (110), (003)
	A Structure: Pbca		
m_a	0	0	1
m_b	1	0	0
m_c	0	1	0
	B Structure: Pb'c'a		
m_a	0	0	0
m_b	0	1	0
m_c	1	0	0

Table S1. Projection of magnetic structures at different reflections

For this reason, we need only measure the resonant and thermal behaviour of three reflections to completely characterise the

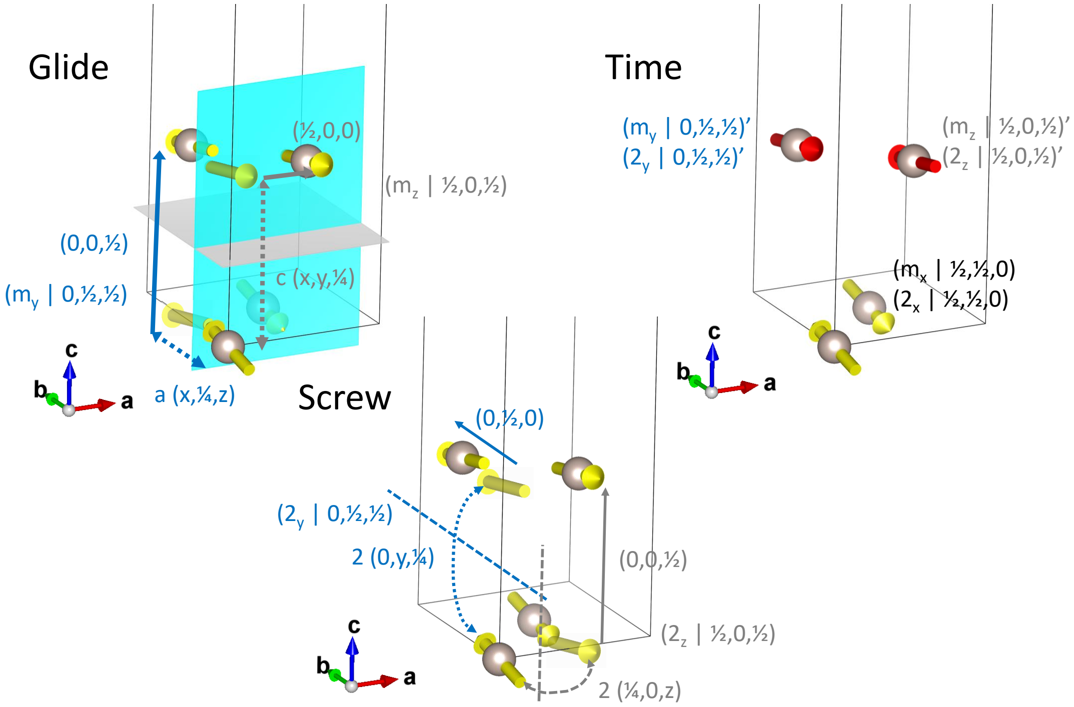

Figure S1. The glide plane, screw axis and time operations are illustrated between the central Ru ion at (0,0,0) and the neighbours in the next layer - Ru(1/2,0,1/2) and Ru(0,1/2,1/2). Note that magnetic moments are axial vectors so transform with inverse parity under mirror operations.

magnetic properties of the system. To avoid the need to re-mount samples along different crystallographic axes, we looked only at the (003), (013) and (103) resonant reflections, using the (004) reflection to normalise for crystal quality between samples and using measurements of the Fluorescent emission to normalise for self-absorption, see Fig. S2. Resonant and fluorescent spectra are corrected for scattering volume and absorption of elements in the beam path, such as the berrylium dome on the cryostat, x-ray windows and air gap. At these low energies this absorption correction is very sensitive, making the extraction of quantitative variations difficult. For this reason we cannot discern any trends in Ru valence or the L3/L2 ratio (see inset of Fig. S2).

The resonant spectra for each reflection below and above the magnetic transition are shown in Fig. S3. As described previously for the un-doped system, the resonant behaviour is a combination of magnetic scattering from the ordered magnetic structure and a non-magnetic part related to the anisotropy of the atomic orbitals – so called anisotropic tensorial scattering (ATS). The magnetic part is much stronger below the magnetic transition temperature and the ATS part potentially exists at all temperatures, though it is minimised at high temperature. The ATS part has previously been attributed to orbital ordering with an apparent transition at $T_{OO} \approx 260$K[2], however we previously ruled-out the possibility of a change in symmetry at this transition and concluded that the increase in signal at this temperature on certain reflections is probably due to a complex phase relation within the (dxz, dyz) manifold[1].

Following the resonant peaks on both reflections as the temperature is decreased, there is a clear difference in behaviour between the pure compound and those with any level of Mn doping, shown in Fig. S4. Magnetic transitions are clear from significant increases in resonant intensity in all of the samples. In each case these changes coincide with the magnetic transitions observed with magnetisation measurements. All samples with Mn doping show a large enhancement on the (103) reflection, consistent with a B-centred magnetic structure. Of these, only the 3% sample shows any change on the (013) reflection to match the behaviour of the pure compound. The magnetic transitions temperatures were fitted from the temperatures and are reported in Table S2 and these confirm a linear increase with Mn concentration.

C Magnetisation

The field-cooled magnetisation of each composition were characterised in both in-plane directions, where the direction was determined by x-ray diffraction. The measured magnetic moment of all compositions, given in Fig. S5, indicate an increasing

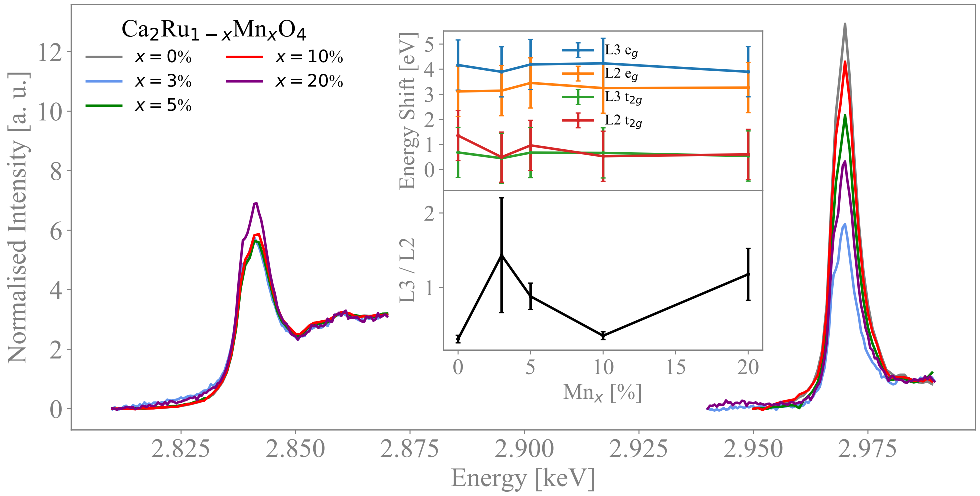

Figure S2. X-Ray absorption spectra measured from fluorescence yield, corrected for scattering volume and for varying absorption in the beam path. Fitted parameters are shown on the inset using a double-Lorentzian profile centered at the t_{2g} and e_g multiplets. The top panel shows the fitted centre of each profile and the bottom panel shows the ratio of areas under each absorption edge.

Mn %	Mag. Structure	T_N
0	A-Centred	106.2 (7)
3	B-Centred	116.14 (2)
5	B-Centred	123.1 (5)
10	B-Centred	135.4 (4)
20	B-Centred	140.2 (2)

Table S2. Transition temperatures of the ordered magnetic phase in different Mn concentrations.

magnetic transition temperature with increasing Mn content. As has been found in other systems[3], the replacement of ruthenium induces a weak ferromagnetic response, seen here by the increased moment at low temperature below the ordering transition. The variation in measured moment at low and high temperature with manganese content is illustrated in Fig. S5(b). The moment increases logarithmically up to 5% but plateaus at higher concentrations, suggesting the ferromagnetic moment is not due to increasing numbers of ferromagnetically ordered Mn ions. In the case of the 3% sample we also see a second transition and a lowering of the moment at a second, lower magnetic transition, such a magnetic response has also been reported when doping with small amounts of Fe[4,5].

D Single Crystal X-Ray Characterisation

Small single crystal samples of each Mn concentration were measured continuously between 90K and 500K using an X-ray diffractometer with liquid nitrogen cryojet. At each temperature, full coverage to a high angle of reciprocal space was obtained and refinements were performed to determine the octahedral bond lengths. The refined structure parameters of each composition are given for 90K and 300K in Table S3 and the temperature dependences for the lattice and atomic structure are shown Figs. S6 and S7 respectively. Refined CIF files are available in the Zenodo repository, 10.5281/zenodo.6497537.

The pure compound is subject to significant structural variations in the atomic bond lengths and distortion angles with temperature, including a negative thermal expansion, particularly along the b-axis. This structural evolution becomes less pronounced as the Mn content is increased, highlighted by the decreasing variation in volume and lattice parameters in Fig S6 (a). The reduction in volume with increased dopant concentration is primarily led by a reduction in the RuO_1 bond length causing decreased flattening of the octahedra combined with a reduction in the octahedral rotation.

The jump in volume at high temperature, normally marked by the insulator-metal transition also becomes significantly less pronounced as manganese is added. This again is also seen in other transition metal doping regimes for CRO, with a flattening of the MIT noted when doping with Sn[6] and the disappearance of the MIT when doing above 10% with Ti[7] and Cr[8]. The flattening of the MIT is however in stark contrast with doping regimes replacing the alkali site, for instance when replacing Ca

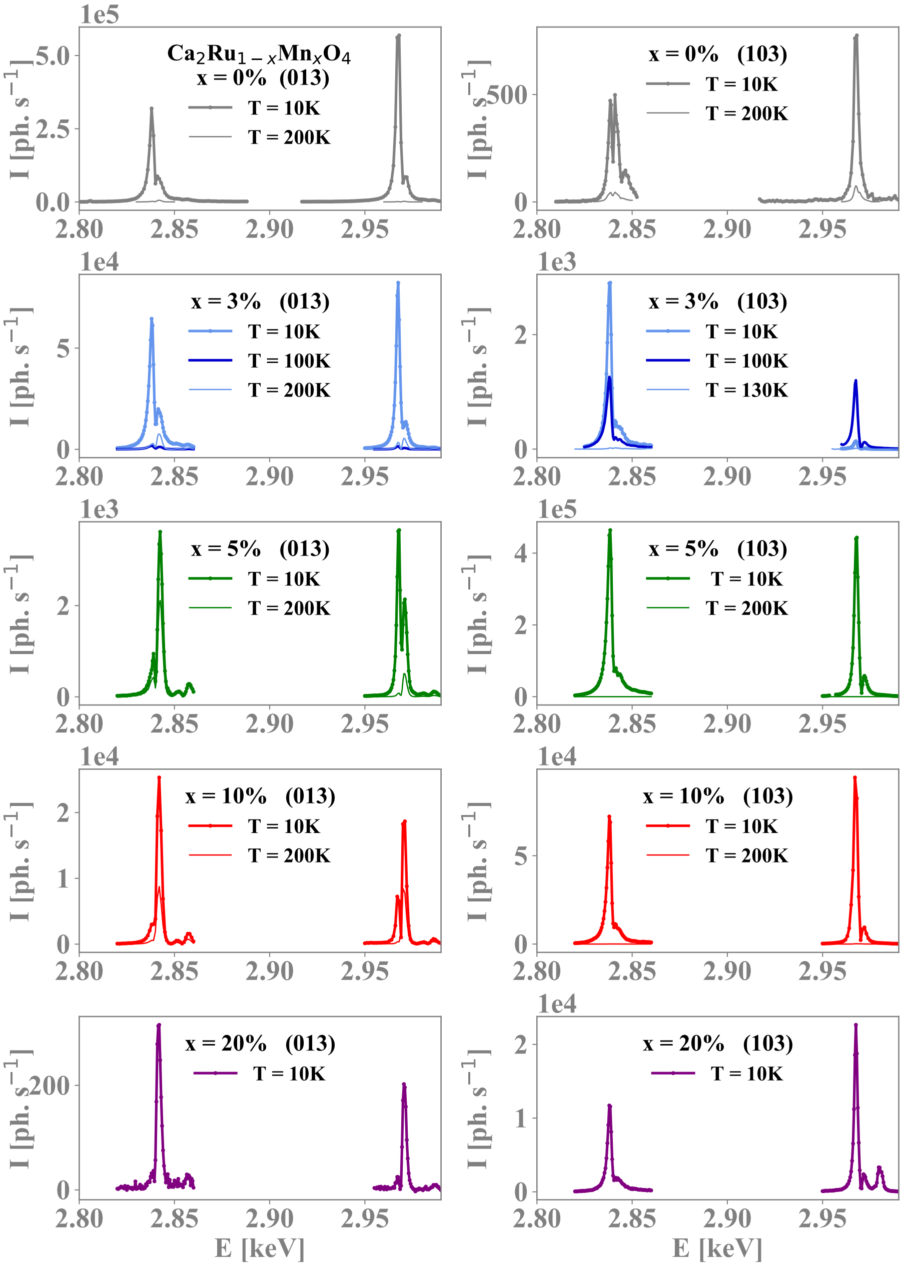

Figure S3. Resonant x-ray spectra at the Ru L_3 and L_2 absorption edge for reflections (013) and (103) at various temperatures. Spectra are normalised by the (004) charge reflection and corrected for self-absorption and the absorption path of the scattered photons including air, beryllium dome and kapton and SiN windows ($I_{L2} \approx 3I_{L3}$).

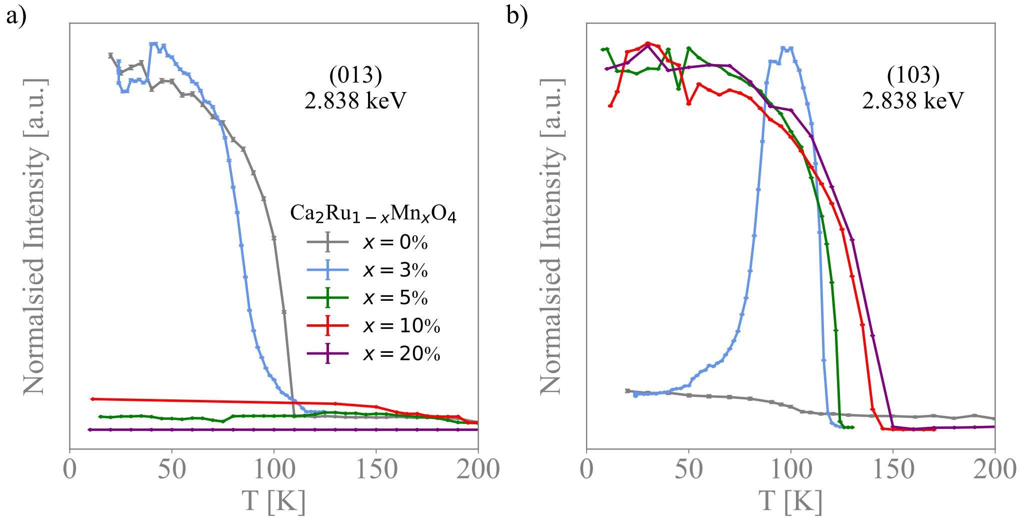

Figure S4. Variation of intensity with temperature at the resonant edge at the a) (013) and b) (103) reflections. The intensity of each dependence is normalised to the maximum magnetic signal in each sample.

with Sr[9,10] or La[11,12], where the structural transition follows T_{MIT} as it decreases.

The magnetic exchange paths in Ca_2RuO_4 lay through the oxygen ions connecting neighbouring octahedra. As illustrated in Fig. 1 of the main article, the shortest exchange path involves a single oxygen along the in-plane diagonal of the structure, with the next nearest neighbours along the in-plane axes a and b, passing two oxygen ions. Only slightly longer is the exchange path along the c-axis also passing two oxygen ions, though in this case the path lengths between neighbouring Ru ions along the a and b axis are quite different. Fig. S8 shows the variation in exchange path with Mn content at different temperatures. Increasing Mn content does not change the thermal evolution of the path lengths but does decrease the in-plane path lengths. Out of plane exchange paths are changed less but are reduced along the b-axis and increased along the a-axis with the difference between pathways along the two directions decreasing as doping is increased.

E Microscopic Model Hamiltonian

We report here the details of the microscopic model describing the energy levels and wave functions of the considered Ru-O_2-O_2-Ru cluster of atoms connected within the (a,c) plane. The examined Hamiltonian is expressed as[13,14]:

$$H = H_{kin} + H_{el-el} + H_{cf} + H_{soc} \,. \tag{1}$$

The first term in Eq. 1 is the kinetic operator describing the Ru-O connectivity:

$$H_{kin} = \sum_{ij,\alpha\beta,\sigma} t_{ij}^{\alpha\beta} \left(p_{i\alpha\sigma}^{\dagger} d_{j\beta\sigma} + h.c.\right) \,, \tag{2}$$

where $d_{i\beta\sigma}^{\dagger}$ is the creation operator for an electron with spin σ at the i site in the β orbital of the t_{2g} sector (d_{xy}, d_{xz}, d_{yz}), while $p_{i\alpha\sigma}$ is the annihilation operator of an electron with spin σ at the i site in the α orbital of the (p_x, p_y, p_z) space of the oxygen. Hopping amplitudes $t_{ij}^{\alpha\beta}$ include all the allowed symmetry terms according to the Slater-Koster rules[15,16] for bonds connecting the two ruthenium via the two oxygen atoms along the $(\frac{1}{2},0,\frac{1}{2})$ direction.

Since we have a low spin configuration for the Ru d^4 electronic state, the local Ru- Hamiltonian H_{lel-el} has the complete Coulomb interaction projected on the t_{2g} electrons, which is expressed in terms of Kanamori parameters U, U', and J_H as follows:

$$H_{el-el} = U \sum_{i\alpha} n_{i\alpha\uparrow} n_{i\alpha\downarrow} - 2J_H \sum_{i\alpha\beta} \mathbf{S}_{i\alpha} \cdot \mathbf{S}_{i\beta} +$$

$$(U' - \frac{J_H}{2}) \sum_{i\alpha\neq\beta} n_{i\alpha} n_{i\beta} + J' \sum_{i\alpha\beta} d_{i\alpha\uparrow}^{\dagger} d_{i\alpha\downarrow}^{\dagger} d_{\mathbf{i}\beta\uparrow} d_{i\beta\downarrow} \tag{3}$$

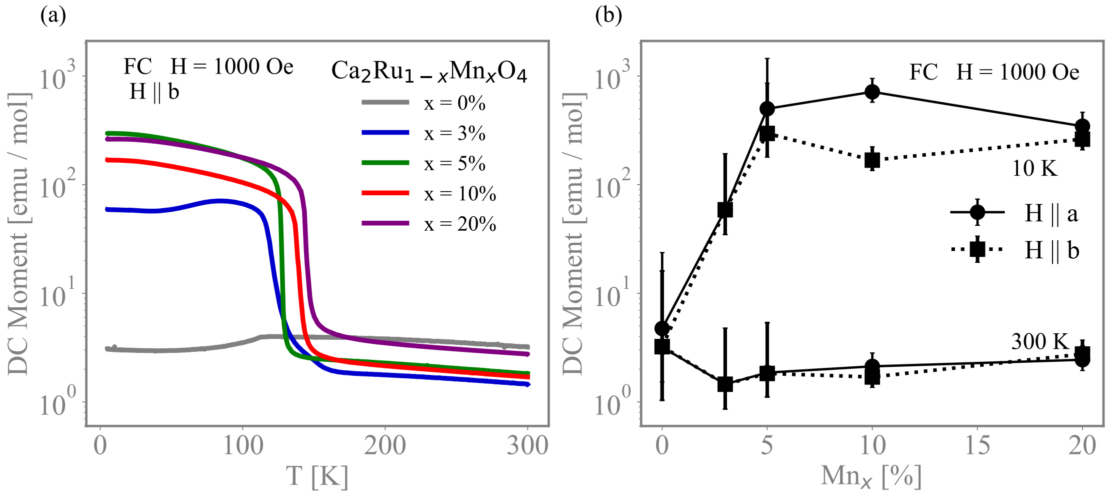

Figure S5. (Colour online) Magnetisation measurements of single crystal samples. a) Measurents on heating in a static field after cooling in zero field, indicating magnetic ordering transitions and a FM moment in the doped samples. The size of the measured molar moment below and above the transition is given in (b).

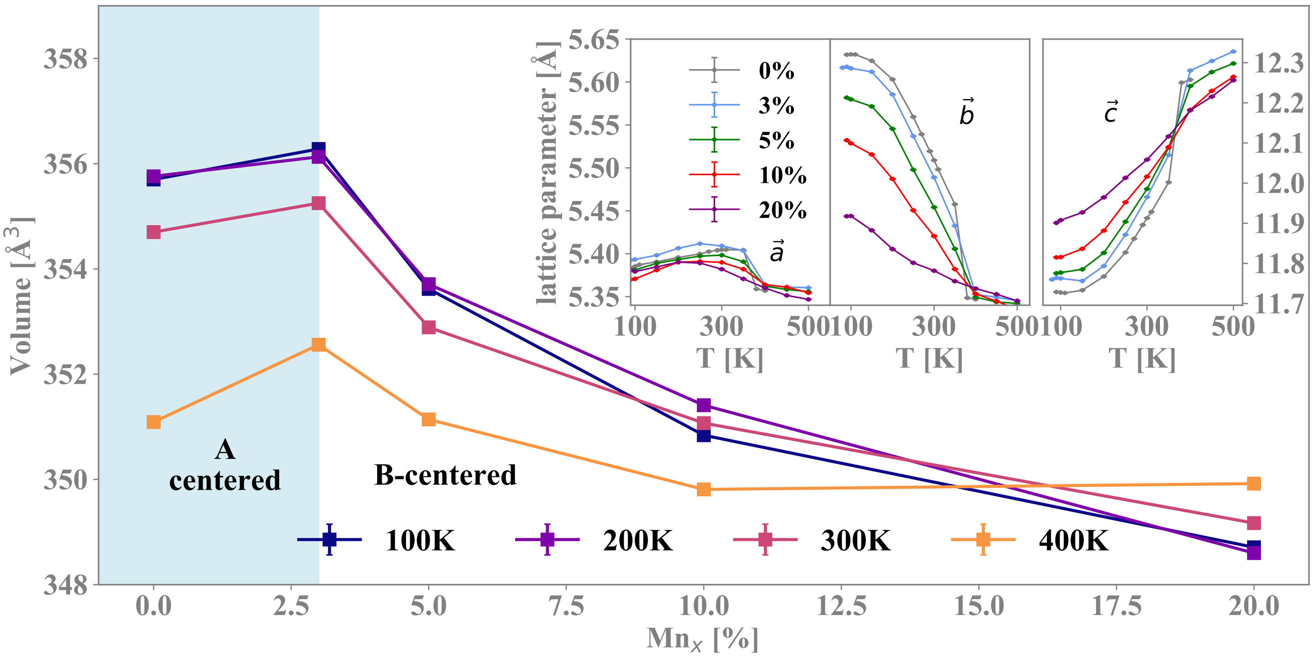

Figure S6. Cell volume of $Ca_2Ru_{1-x}Mn_xO_4$ as a function of Mn concentration (x) and temperature (T), determined by x-ray diffraction. The thermal evolution of the lattice parameters of each composition are given in the inset.

		x = 0.03		x = 0.05		x = 0.10		x = 0.20	
		90K	300K	90K	300K	90K	300K	90K	300K
	a	5.3924(3)	5.4091(3)	5.3807(3)	5.3983(3)	5.3698(3)	5.3899(3)	5.3813(3)	5.3820(3)
	b	5.6177(4)	5.4889(4)	5.5818(4)	5.4541(4)	5.5322(4)	5.4205(4)	5.4436(4)	5.3802(4)
	c	11.7639(8)	11.9652(8)	11.7754(8)	11.9855(8)	11.8150(8)	12.0163(8)	11.9010(8)	12.0585(8)
	Volume	356.36(4)	355.25(4)	353.66(4)	352.89(4)	350.99(4)	351.07(4)	348.62(4)	349.17(4)
	ΔV/V	-0.31%		-0.22%		-0.02%		+0.16%	
Mn	occ.	0.026(10)	0.000(5)	0.070(3)	0.059(3)	0.122	0.119(3)	0.173(10)	0.176(3)
Ca	x	0.00330(10)	0.00802(5)	0.00461(3)	0.00915(3)	0.00616(6)	0.00967(5)	0.00689(11)	0.00958(5)
	y	-0.05744(10)	-0.04414(7)	-0.05546(4)	-0.04171(6)	-0.05121(6)	-0.03741(6)	-0.04134(17)	-0.03196(6)
	z	0.35240(5)	0.35095(3)	0.352354(19)	0.35081(2)	0.35205(3)	0.35063(2)	0.35153(5)	0.350460(16)
O1	x	0.3054(4)	0.3032(2)	0.30462(14)	0.30246(13)	0.3041(2)	0.30276(19)	0.3031(6)	0.30253(16)
	y	0.1987(4)	0.1986(2)	0.19901(13)	0.19841(14)	0.1990(2)	0.19821(19)	0.1988(6)	0.19783(17)
	z	0.0270(2)	0.02314(12)	0.02641(7)	0.02235(7)	0.02493(11)	0.02082(9)	0.0225(3)	0.01831(8)
O2	x	-0.0681(4)	-0.0581(2)	-0.06588(17)	-0.05534(18)	-0.0632(3)	-0.0521(2)	-0.0561(6)	-0.0465(2)
	y	0.0217(4)	0.0169(3)	0.02072(14)	0.01574(16)	0.0200(2)	0.0141(2)	0.0175(7)	0.01187(19)
	z	0.1645(2)	0.16477(11)	0.16438(7)	0.16465(7)	0.16441(10)	0.16463(8)	0.1641(3)	0.16428(7)
	RuO1	2.015(2)	1.9887(12)	2.0043(8)	1.9771(8)	1.9914(12)	1.9697(10)	1.976(3)	1.9577(9)
	RuO2	1.973(3)	1.9985(13)	1.9712(9)	1.9978(9)	1.9750(13)	1.9995(10)	1.978(3)	1.9977(9)
	O1RuO1	90.86(9)	89.72(5)	90.66(3)	89.52(3)	90.41(5)	89.41(4)	89.63(13)	89.27(4)
	O1RuO2	87.90(10)	88.11(6)	87.83(3)	88.06(4)	87.96(5)	88.27(4)	88.06(13)	88.57(4)
	Refs.	3022	3014	2561	2587	1481	1475	3111	2938
	R_w(all)	0.0827	0.0393	0.0402	0.0342	0.0234	0.0226	0.1011	0.0290

Table S3. Refined structural parameters from single crystal XRD measurements. RuO1 and RuO2 distances are in Å. O1 and O2 refer to the planar and apical oxygen positions, respectively. CIF files with full refinement details and refined thermal parameters are available in the supplementary material.

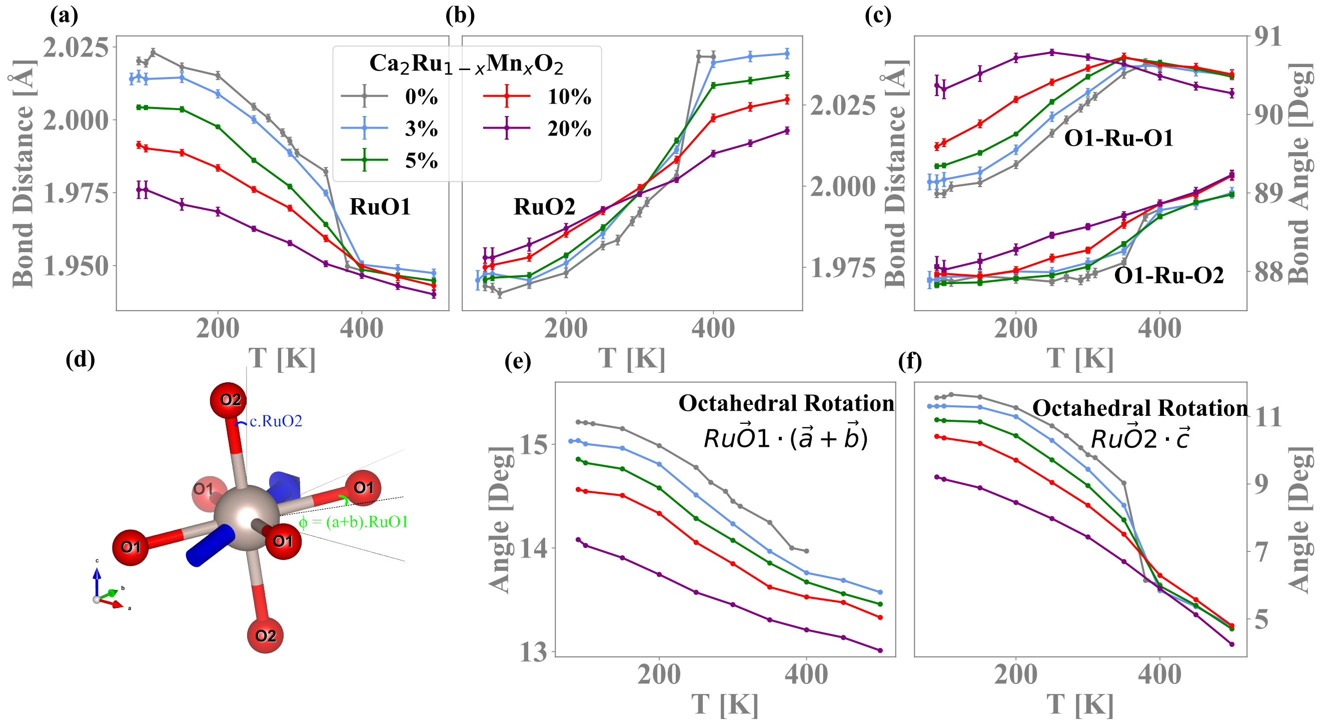

Figure S7. Variation with temperature of refined structure parameters from single crystal XRD measurements. Panels (a) and (b) show the change in planar Ru-O1 and apical Ru-O2 bond distance, respectively. Panel (c) gives the internal bond angles within the octahedra. The octahedral distortions angles, as defined in panel (d), are shown in panels (e) and (f).

where $n_{i\alpha\sigma}$, $\mathbf{S}_{i\alpha}$ are the on site charge density for spin σ and the spin operators for the α orbital, respectively. U (U') is the intra (inter)- orbital Coulomb repulsion, J_H is the Hund's coupling, and J' the pair hopping term. Due to the invariance for rotations in the orbital space, the following relations hold: $U = U' + 2J_H$, $J' = J_H$.
The H_{cf} part of the Hamiltonian H is the crystalline field potential (CF), controlling the symmetry lowering from cubic to tetragonal one, due to the compression of RuO_6 octahedra along the c axis.:

$$H_{cf} = \delta \sum_{i=1,2} [n_{ixy} - \frac{1}{2}(n_{izx} + n_{iyz})] \tag{4}$$

Finally, we include the the spin-orbit coupling Hamiltonian

$$H_{soc} = \lambda \sum_i \mathbf{L}_i \cdot \mathbf{S}_i \,. \tag{5}$$

Due to the cubic CF terms in RuO_6 octahedra separating the lower t_{2g} from the unoccupied e_g levels, $\mathbf{L_i}$ stands for the angular momentum operator projected onto the t_{2g} subspace. Its components have the following expression in terms of orbital fermionic operators:

$$\begin{aligned} L_{ix} &= i \sum_\sigma [d^\dagger_{ixy\sigma} d_{izx\sigma} - d^\dagger_{izx\sigma} d_{ixy\sigma}] \\ L_{iy} &= i \sum_\sigma [d^\dagger_{iyz\sigma} d_{ixy\sigma} - d^\dagger_{ixy\sigma} d_{iyz\sigma}] \\ L_{iz} &= i \sum_\sigma [d^\dagger_{izx\sigma} d_{iyz\sigma} - d^\dagger_{iyz\sigma} d_{izx\sigma}] \end{aligned} \tag{6}$$

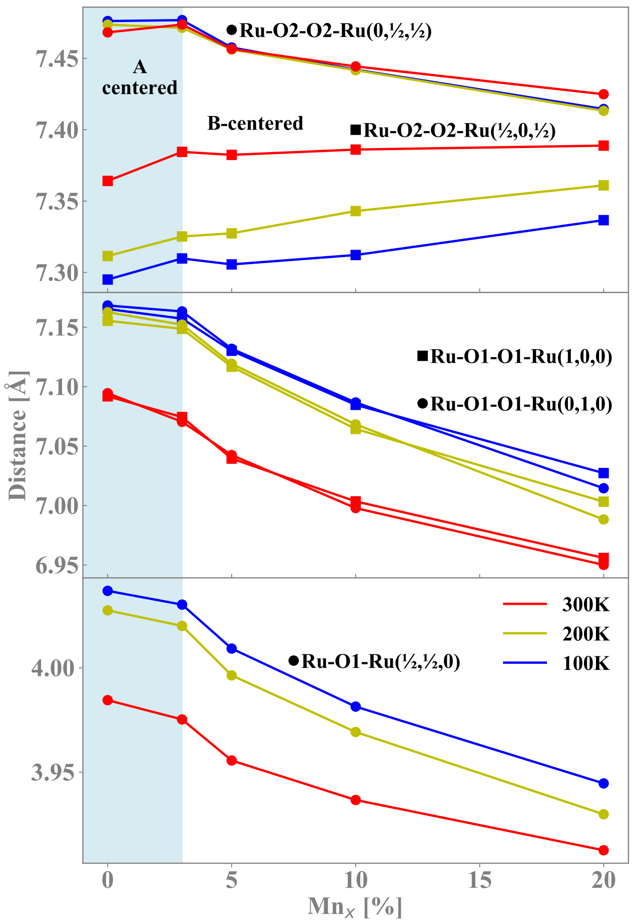

Figure S8. (Colour online) Variation in exchange path distance between neighbouring Ru ions with Mn content, determined using atomic positions from x-ray refinements. The Ru exchange paths are depicted in Fig. 1 (a) of the main paper.

References

1. Porter, D. G. *et al.* Magnetic anisotropy and orbital ordering in ca_2ruo_4. *Phys. Rev. B* **98**, 125142, DOI: 10.1103/PhysRevB.98.125142 (2018).
2. Zegkinoglou, I. *et al.* Orbital ordering transition in Ca2RuO4 observed with resonant X-ray diffraction. *Phys. Rev. Lett.* **95**, 136401, DOI: 10.1103/PhysRevLett.95.136401 (2005).
3. Autieri, C. Antiferromagnetic andxyferro-orbital order in insulating SrRuO3thin films with SrO termination. *J. Physics: Condens. Matter* **28**, 426004, DOI: 10.1088/0953-8984/28/42/426004 (2016).
4. Qi, T. F., Korneta, O. B., Parkin, S., Hu, J. & Cao, G. Magnetic and orbital orders coupled to negative thermal expansion in mott insulators $ca_2ru_{1-x}M_xo_4$ (m = mn and fe). *Phys. Rev. B* **85**, 165143, DOI: 10.1103/PhysRevB.85.165143 (2012).
5. Chi, S., Ye, F., Cao, G., Cao, H. & Fernandez-Baca, J. A. Competition of three-dimensional magnetic phases in $ca_2ru_{1-x}fe_xo_4$: A structural perspective. *Phys. Rev. B* **102**, 014452, DOI: 10.1103/PhysRevB.102.014452 (2020).
6. Takenaka, K. *et al.* Extended operating temperature window of giant negative thermal expansion in sn-doped ca2ruo4. *Appl. Phys. Lett.* **113**, 071902, DOI: 10.1063/1.5046463 (2018).
7. Kunkemöller, S. *et al.* Magnon dispersion in Ca 2 Ru 1 - x Ti x O 4: Impact of spin-orbit coupling and oxygen moments. *Phys. Rev. B* **95**, 214408, DOI: 10.1103/PhysRevB.95.214408 (2017).
8. Qi, T. F. *et al.* Negative volume thermal expansion via orbital and magnetic orders in $ca_2ru_{1-x}cr_xo_4(0 < x < 0.13)$. *Phys. Rev. Lett.* **105**, 177203, DOI: 10.1103/PhysRevLett.105.177203 (2010).
9. Nakatsuji, S. & Maeno, Y. Switching of magnetic coupling by a structural symmetry change near the Mott transition in Ca2-xSrxRuO4. *Phys. Rev. B* **62**, 6458–6466, DOI: 10.1103/PhysRevB.62.6458 (2000).
10. Friedt, O. *et al.* Structural and magnetic aspects of the metal-insulator transition in Ca 2 - x Sr x RuO 4. *Phys. Rev. B* **63**, 174432, DOI: 10.1103/PhysRevB.63.174432 (2001).
11. Cao, G. *et al.* Ground-state instability of the mott insulator ca_2ruo_4 : impact of slight la doping on the metal-insulator transition and magnetic ordering. *Phys. Rev. B* **61**, R5053–R5057, DOI: 10.1103/PhysRevB.61.R5053 (2000).
12. Fukazawa, H. & Maeno, Y. Filling control of the mott insulator ca 2ruo 4. *J. Phys. Soc. Jpn.* **70**, 460–467, DOI: 10.1143/JPSJ.70.460 (2001).
13. Cuoco, M., Forte, F. & Noce, C. Probing spin-orbital-lattice correlations in 4 d 4 systems. *Phys. Rev. B* **73**, 094428, DOI: 10.1103/PhysRevB.73.094428 (2006).
14. Cuoco, M., Forte, F. & Noce, C. Interplay of Coulomb interactions and. *Phys. Rev. B* **74**, 195124, DOI: 10.1103/PhysRevB.74.195124 (2006).
15. Slater, J. C. & Koster, G. F. Simplified lcao method for the periodic potential problem. *Phys. Rev.* **94**, 1498–1524, DOI: 10.1103/PhysRev.94.1498 (1954).
16. Brzezicki, W., Noce, C., Romano, A. & Cuoco, M. Zigzag and checkerboard magnetic patterns in orbitally directional double-exchange systems. *Phys. Rev. Lett.* **114**, 247002, DOI: 10.1103/PhysRevLett.114.247002 (2015).
